# Induction of Labor: A Narrative Review on Cost Efficiency in Maternity Care

**DOI:** 10.7759/cureus.71302

**Published:** 2024-10-12

**Authors:** Alaa Mamieh, Kritanjali Saha, Saya Alasaadi, Shayla L Holman, Flavio Veintemilla-Burgos, Lucia Santistevan, Shama Rani Paul, Maria Kamel, Pinima Godpower, Lasya Reddy Pesaru

**Affiliations:** 1 Medicine, University of Georgia, Tbilisi, GEO; 2 Medicine, Smt. Nathiba Hargovandas Lakhmichand (NHL) Municipal Medical College, Gujarat, IND; 3 Medicine, University College Dublin, Dublin, IRL; 4 Medicine, St. George’s University School of Medicine, True Blue, GRD; 5 Medical Sciences, Universidad Católica de Santiago de Guayaquil, Guayaquil, ECU; 6 Medical Sciences, Universidad de San Martin de Porres, Lima, PER; 7 Medicine, MAHSA (Malaysian Allied Health Sciences Academy) University, Selangor, MYS; 8 Medicine, Columbus Central University School of Medicine, Ladyville, BLZ; 9 Medicine, Kamineni Institute of Medical Sciences, Narketpally, IND

**Keywords:** cesarean section, cost-efficiency, healthcare management, labor induction, maternity care, vaginal delivery

## Abstract

The clinical and financial implications of induction of labor (IOL) in comparison to elective cesarean sections and expectant management are examined in this review. IOL is frequently used to avoid complications such as hypertensive disorders and stillbirth, but is can be expensive, particularly if a failed induction is followed by a cesarean. The cost-effectiveness of IOL varies based on factors such as gestational age, maternal obesity, and prior cesareans. Misoprostol has proven to be a more cost-effective induction method than oxytocin, with higher success rates for vaginal delivery and shorter hospital stays. However, spontaneous labor remains the most cost-efficient option, requiring fewer interventions and reducing costs. Membrane sweeping is one alternative that reduces costs and promotes unplanned labor. Although high-risk situations may necessitate elective IOL, routine use of these devices without a medical necessity raises expenditures without enhancing outcomes. It is recommended that healthcare professionals implement careful labor management techniques, utilizing cost-effective approaches whenever feasible, particularly in resource-limited settings. More randomized trials are required to evaluate the long-term effects of IOL on costs and health, thus shaping future labor management strategies.

## Introduction and background

Induction of labor (IOL) is usually described as encouraging the uterus to contract before labor naturally starts [1]. It is occasionally used to induce delivery vaginally. The basic reason behind IOL concerns the health of the baby or the pregnant mother. IOL is primarily advised if it is anticipated that giving birth would result in better results for the mother and newborn than extending the pregnancy; intrauterine growth limitation, post-term pregnancy, hypertensive problems, and elective reasons are the most prevalent causes of IOL [2,3]. This is unlike the 18th and 19th centuries when pelvic deformity was the major reason for considering IOL [4]. A considerable increase in the trend of IOL has been observed over the past decade, especially in high-income countries like Australia, Europe, and the United States [5,6]. 

An increase in the incidence of IOL has been observed through the past decade and this can sometimes be attributed to medical complications related to pregnancy or may be opted by the patient despite normal pregnancy and health. The percentage of cesarean section deliveries grew from 2.7% to 4.5% between 1958 and 1970, as did the rate of forceps deliveries, which increased from 4.7% to 7.9% [7]. Moreover, IOL has also been associated with premature births and reduced maternal satisfaction, along with a probable intervention cascade [4]. Possible reasons for the rise in IOL are a significant decrease in perinatal mortality, shorter labor, healthier newborns, and safety for mothers [7]. A recent study has reported that the rate of cesarean sections among nulliparous women undergoing induced labor rose from 14.0% in those under 20 years of age to 39.9% in those over 40 [8]. The primary method of inducing labor is to artificially trigger the labor and delivery process which can be achieved by pharmacological, non-pharmacological, mechanical, and complementary methods [1]. Current literature explores different techniques and timing of labor induction, including the specific circumstances under which it is performed, along with the maternal care provided during the process. [9]. The pharmacological methods mainly comprise intravaginal or cervical prostaglandins, oxytocin, amniotomy, oxytocin combined with amniotomy, oral misoprostol, vaginal misoprostol, and buccal or sublingual misoprostol. Non-pharmacological methods include the use of Foley catheters and balloon catheters. Complementary methods like the use of castor oil or acupuncture have been less popular due to their minimal effectiveness and limited use [10]. The likelihood of complications and childbirth varies with the type of method opted for IOL. 

The direct and indirect expenses of various induction techniques vary, and some include continuous labor monitoring. Evidence implies that, in contrast to expectant management, inducing labor in women with complications is linked with lower health service expenditures. On the other hand, the costs of one induction technique over another are not well-established [11]. While looking at the cost-effectiveness of IOL at 41 weeks of gestation reported that it proved to be successful along with a significant decline in adverse obstetric outcomes such as neonatal demise, perineal lacerations, shoulder dystocia, and meconium aspiration syndrome [12]. Another study that compared outpatient labor induction with a balloon catheter to inpatient prostaglandin vaginal gel stated that while resulting in similar health outcomes, outpatient labor induction proved to be more cost-effective than the latter [13]. IOL has also proved to be a cost-effective and safer option in cases of fetal macrosomia/large gestational age to avoid severe complications like postpartum hemorrhage, birth trauma, and maternal or neonatal morbidity [14]. The primary purpose of this narrative review is to provide a thorough understanding of the IOL approach through an analysis and summary of the substantial amount of evidence on mother care and cost-effectiveness.

## Review

Indication for IOL

While reducing morbidity and mortality can be greatly aided by inducing labor, the benefits of this intervention are at best minimal or perhaps unknown, and an increasing number of women (and their children) are being exposed to the drawbacks of this procedure. A growing number of factors are being evaluated as indicators of the need to induce labor, including advanced maternal age, non-native ethnicity, high BMI, artificially aided conception, and even nulliparity [15].

The obstetrical and medical history of a patient determines the criteria for late preterm, early term, late-term, and post-term timing of birth. When it is believed that the outcomes for the woman, the fetus, or both are preferable to expectant management, that is, waiting for the labor to start on its own, IOL is recommended [16].

The American College of Obstetricians and Gynecologists (ACOG) provides specific delivery timing recommendations based on clinical scenarios, such as intrauterine growth restriction (IUGR), hypertension, preeclampsia, diabetes, and other conditions like preterm premature rupture of the membranes (PPROM), abruptio placentae, and intrauterine fetal demise (IUFD). Delivery timing ranges from as early as 32 weeks for severe cases to 41 weeks for late-term pregnancies [3].

Logistics-related factors such as the likelihood of early labor, the distance to the hospital, or psychological signs, can also lead to the IOL. Fetal lung maturity should be determined in these situations. A mature fetal lung test result obtained before 39 weeks of gestation is not a basis for delivery in the absence of suitable clinical circumstances [17].

In 20% of pregnancies, IOL is carried out. But little research has been done on how the induction indications affect the failure rate of the device. A study revealed that induction failure occurred more frequently in nulliparous women (p=0.0001), high maternal age (p = 0.047), and at an earlier gestational age (p=0.0001). In women who were nulliparous, maternal indications had a significant correlation with both cesarean delivery (adjusted odds ratio (aOR) 2.36, 95%CI 0.40-2.29, p = 0.019) and induction failure (aOR 2.52, 95%CI 1.28-4.95, p = 0.007). Parental indications (aOR 4.22, 95%CI 1.14-15.58, p = 0.031) and hypertensive problems (aOR 7.26, 95%CI 1.89-27.87, p = 0.004) among multiparous women were strongly linked with induction failure, but not cesarean delivery. The research findings indicate that the decision to induce labor may have an effect on the chance of failure [18].

Disadvantages and complications associated with IOL

IOL is a common intervention used in obstetric practice, but it is not without risks. Several studies have examined the complications associated with different methods of IOL, and the findings underscore the need for careful consideration of risks, particularly in specific populations such as obese women or in cases involving certain induction methods. Maternal obesity has been shown to increase the risk of complications following IOL, particularly in cases of prolonged pregnancy. A study conducted by Arrowsmith et al. demonstrated that obese women had a significantly higher rate of cesarean section following IOL compared to women of normal weight (38.7% versus 23.8% in primiparous women) [19]. This increased risk is likely due to difficulties in achieving effective cervical ripening and progression of labor in obese women. Despite the higher rate of cesarean sections, other labor-related complications such as postpartum hemorrhage and third-degree tears were similar between obese and non-obese women. The study concluded that while maternal obesity increases the likelihood of cesarean section following IOL, it remains a safe management option in prolonged pregnancies, as most obese women still achieved vaginal delivery.

Pharmacological agents used for IOL, such as misoprostol, are associated with complications like uterine hyperstimulation. According to a review by Alhazmi et al., vaginal misoprostol (≥50 µg) was found to significantly increase the odds of excessive uterine activity, which can lead to fetal distress and other maternal complications [20]. In contrast, the use of a double-balloon catheter was shown to have the highest probability of being among the safest methods with fewer complications like uterine hyperstimulation. These findings suggest that while pharmacological agents are effective, their use requires close monitoring due to the potential for adverse outcomes.

Boulvain et al. investigated the risks associated with IOL in uncomplicated term pregnancies and found that it was linked to a higher risk of cesarean section (RR=2.4) compared to spontaneous labor [21]. The study also highlighted an increased use of epidural and non-epidural analgesia in induced labor. Additionally, perinatal complications such as the need for resuscitation, admission to the intensive care unit, and phototherapy were more frequent following IOL. These results emphasize that while IOL is often necessary for managing certain pregnancies, it should be carefully considered in low-risk cases due to the increased risk of cesarean section and perinatal complications.

The transcervical balloon catheter is frequently used for cervical ripening in IOL and has been associated with a relatively low risk of complications. However, a systematic review by Gommers et al. found that maternal intrapartum infections occurred in 11.3% of cases, with a postpartum infection rate of 3.3% [22]. The review also reported that uterine hypercontractility occurred in 2.7% of women, while neonatal complications such as non-reassuring fetal heart rate and meconium-stained liquor were present in 10.8% and 14.0% of cases, respectively. Although the balloon catheter is considered a safe option, these findings indicate that it is not without risks, particularly in terms of infections and neonatal distress.

Current clinical practices and economic implications

IOL vs. Expectant Management

IOL and expectant management are two key strategies used in obstetric care. IOL is often recommended when the benefits outweigh the risks, particularly in high-risk pregnancies, while expectant management allows labor to begin spontaneously. Studies show that elective IOL at 39-41 weeks can reduce the risk of complications such as stillbirth, hypertensive disorders, and shoulder dystocia [12,23]. However, this comes at an increased cost. For example, induction at 39 weeks leads to increased healthcare expenditures, largely driven by prolonged labor and higher cesarean section rates [24,25]. On the other hand, expectant management has shown lower short-term costs but higher risks of stillbirth and neonatal complications [26]. Thus, while IOL may prevent adverse outcomes, its financial burden on the healthcare system is significant.

Economic Burden of Cesarean Sections

Cesarean deliveries, particularly those following failed inductions, carry higher costs than vaginal births. Studies such as those by Garcia-Simon et al. have reported that cesarean deliveries after IOL cost significantly more (€4830.45) compared to spontaneous vaginal deliveries (€3037.45) [27]. This economic burden increases in cases with an unfavorable cervix, where induction often leads to a cesarean section. Additionally, scheduled cesarean deliveries, though costly, might prevent the need for emergency interventions, thus balancing costs in high-risk cases [28]. Planned cesareans in specific populations, such as women with class III obesity, can also present a cost-effective option compared to induction [29].

Cost-effectiveness of different IOL methods 

Comparing IOL Techniques (e.g., Misoprostol, Oxytocin)

Various induction methods have differing levels of cost-effectiveness and clinical efficacy. Misoprostol has emerged as one of the more cost-effective options for IOL, particularly for routine vaginal deliveries, which are generally less expensive than instrumental deliveries or cesarean sections [30]. 

According to the literature, using misoprostol more frequently can result in significant cost savings because it shortens hospital stays and lowers the incidence of complications [13]. Despite being widely utilized, oxytocin tends to require more resources because of the increased incidence of uterine hyperstimulation and the procedures that follow [26].

Maternal and Neonatal Outcomes in Relation to Costs

The cost-effectiveness of IOL is directly impacted by clinical outcomes such as the rate of cesarean sections, maternal infections, and newborn problems. Research has indicated, for example, that the induction of labor in morbidly obese women is associated with increased incidence of surgical site infections and readmissions to the hospital, hence raising overall expenditures [28]. In a similar vein, while expectant care increases the likelihood of maternal problems like hypertensive disorders, IOL lowers that likelihood, which can offset the related expenses [31]. The IOL group has better neonatal outcomes, such as fewer respiratory problems and neonatal intensive care unit (NICU) admissions, which increases its cost-effectiveness [25].

Factors influencing cost-effectiveness

Patient-Specific Factors

Several patient-specific factors influence the cost-effectiveness of IOL, including maternal obesity, gestational age, and previous cesarean deliveries. Obese women undergoing IOL are more likely to experience prolonged labor and increased cesarean rates, driving up costs [27]. Additionally, the gestational age at induction plays a critical role, as IOL at 41 weeks is considered more cost-effective in reducing neonatal and maternal complications compared to expectant management up to 42 weeks [26]. Women with a history of cesarean delivery may benefit more from scheduled cesareans, as repeat cesareans following failed inductions lead to higher healthcare costs [29].

Healthcare System Variables

The cost-effectiveness of IOL is also shaped by healthcare infrastructure and resource availability. In high-resource settings, where healthcare systems can handle the increased demands of labor induction, IOL tends to be more economically viable due to better management of complications and shorter hospital stays [24]. However, in low-resource settings, expectant management might be a more feasible option due to the lower immediate costs and the reduced need for medical interventions [32]. National health policies also play a role, as guidelines for labor induction vary widely, impacting how resources are allocated and managed in different healthcare systems [33].

Sensitivity Analyses and Economic Thresholds

Sensitivity analyses have shown that small changes in variables such as the cost of induction or the rate of cesarean deliveries can significantly impact the cost-effectiveness of IOL. For instance, Hersh et al. found that an increase in the cost of induction by $180 would render elective induction at 39 weeks no longer cost-effective [23]. Similarly, the cost-effectiveness of IOL decreases if cesarean rates following induction exceed 57%, highlighting the need for careful patient selection [29]. These findings underscore the importance of considering clinical and economic variables when evaluating the overall cost-effectiveness of IOL. 

Table 1 summarizes the cost-effectiveness and clinical outcomes associated with different IOL methods and comparisons to other labor management strategies. Most studies show that IOL can be cost-effective in specific contexts, though outcomes such as reduced cesarean rates and improved health vary across methods and populations.

**Table 1:** Summary of findings on cost-effectiveness and clinical outcomes of induction of labor (IOL) across various studies

Authors, year of study	Study Location	Study Design	Results	Cost-effective	Improves outcomes
Kaimal et al., 2011 [12]	United States	Decision analytic model	IOL is more cost-effective with fewer adverse outcomes.	Yes	Yes
Hersh et al., 2019 [23]	United States	Cost-effectiveness model	Fewer cesarean deliveries, hypertensive disorders, but costlier.	No	Yes
Hopkins et al., 2019 [28]	United States	Cost analysis of IOL versus scheduled Cesarean Delivery	IOL is cost-saving unless cesarean rate >70%.	Yes	Yes
Cowett et al., 2006 [34]	United States	Decision analysis model	Dilation is cheaper and more effective than IOL.	No	No
Schmidt et al., 2021 [25]	United States	Decision analysis model	Fewer cesareans, less hypertensive disorders, but not cost-effective.	No	Yes
Bruinsma et al., 2023 [26]	Netherlands	Economic evaluation part of INDEX trial	Cost-effective for nulliparous, not for multiparous women.	Yes for nulliparous while No for multiparous	Yes
Hersh et al., 2020 [31]	United States	Retrospective cohort study	Higher maternal hospitalization costs for IOL; lower neonatal costs.	No	Not conclusive
Poinas et al., 2022 [30]	-	Modeling study and literature review	More routine vaginal deliveries, cost-saving.	Yes	Yes
Merollini and Beckmann, 2021 [13]	Australia	Economic evaluation	Outpatient balloon is more cost-effective than inpatient prostaglandin.	Yes	No difference
Lakić et al., 2014 [35]	Serbia	Decision model	Induced vaginal labor is cheaper than emergency cesarean.	Yes	Yes
Einerson et al., 2020 [36]	Utah	Economic analysis	No cost difference, but higher inpatient costs with IOL.	No	Not explored
Garcia-Simon et al., 2016 [27]	Spain	Prospective study	IOL costs €3589, cesarean is more expensive at €4830.	Yes	Not explored
Beckmann et al., 2016 [37]	-	Randomized controlled trial and decision analytic model	Early amniotomy saves costs over repeated prostaglandin.	Yes	No difference
Washburn et al., 2021 [38]	-	Retrospective study	Outpatient inductions reduce hospitalization time and costs.	Yes	Yes
Subramaniam et al., 2016 [29]	-	Cost-minimization analysis based on retrospective cohort	IOL is slightly cheaper, more cesareans with IOL.	Yes	No
Bierut et al., 2016 [33]	Austria, Poland, Romania, Russia and Slovakia	Retrospective study	Reduced hospital staff time, shorter stay, and lower total costs with MVI.	Yes	No difference
Vijgen et al., 2014 [32]	Netherlands	Economic analysis based on a randomized clinical trial	No significant reduction in neonatal sepsis, higher costs for IOL.	No	No difference
Kaufman et al., 2002 [39]	-	Decision-tree model	Induction never cost-saving, but less expensive at later gestational ages.	No	No
Saunders et al., 2022 [40]	United States	Theoretical, cost-consequence model	Lower costs and nurse time compared to prostaglandins, improved results in nulliparous women.	Yes	Yes
Allen et al., 2005 [41]	Nova Scotia	18-year population-based cohort study	Cesarean in labor is more expensive than spontaneous or assisted vaginal delivery, higher costs for IOL.	No	Not mentioned
Farmer et al., 1996 [42]	United States	Cohort study	Outpatient group incurred lower costs and hospital stay, fewer differences in failed inductions or complications.	Yes	No difference

Discussion 

In addition to the clinical implications of IOL, the study by Garcia-Simon et al. focuses on the economic impact of IOL, providing insight into the significant costs associated with different delivery methods [27]. The study found that the mean total cost of IOL was €3589.87, with cesarean deliveries after induction being significantly more expensive (€4830.45) compared to spontaneous vaginal delivery (€3037.45) and instrumental vaginal delivery (€3344.31) [27]. According to the study, there can be a significant financial burden associated with IOL because of the way that poor cervical abnormalities at admission and obstetric complications including hypertensive disorders drive up expenditures. The economic perspective emphasizes the significance of carefully considering induction decisions due to the higher costs associated with cesarean deliveries following induction, in contrast to the findings of Sotiriadis et al., which favored elective induction at 39 weeks for its clinical benefits [43]. The advantages of avoiding needless inductions, however, are emphasized by Chauhan et al. [44]. Methods like membrane sweeping can encourage spontaneous labor and lessen the financial and medical costs associated with formal IOL. When choosing an IOL, this holistic viewpoint emphasizes the necessity to weigh therapeutic outcomes against financial factors.

Studies on the cost-effectiveness of IOL show that various outcomes are possible, many of which rely on the particular clinical situation and patient. Studies by Hersh et al. [31] and Schmidt et al. [25] demonstrate that the overall cost-benefit ratio is frequently unfavorable, especially when cesarean rates are high. However, IOL can be cost-effective in some circumstances, such as lowering poor outcomes at 41 weeks [12].

Misoprostol has been noted as more cost-effective compared to other methods, such as oxytocin, due to its lower associated costs and higher rates of successful vaginal delivery [30]. However, induction methods like balloon catheters have also been shown to provide cost savings when used in outpatient settings [13]. When comparing IOL to alternatives such as elective cesarean sections, the economic burden remains higher for IOL, particularly in cases where inductions fail, leading to emergency interventions [24,27]. In contrast, Cowett et al., in their study, emphasize that dilation and evacuation procedures for second-trimester pregnancy termination are more cost-effective than IOL, highlighting how the context and patient population influence cost-effectiveness [34]. Similarly, elective cesareans, particularly in high-risk populations like women with obesity, may present a more economical choice than IOL, avoiding the risk of prolonged labor and higher intervention rates [29]. Thus, the decision to induce labor should be tailored to individual patient factors and cost-effectiveness considerations, particularly in high-risk pregnancies.

Several gaps in the current research must be acknowledged. Many studies are based on decision-analysis models or retrospective cohort analyses, which may not capture the full range of clinical and economic outcomes associated with IOL in diverse populations. Moreover, there is a lack of comprehensive data from low-resource settings where healthcare infrastructure might significantly impact the cost-effectiveness of IOL. For example, Cowett et al. point out that most studies evaluating IOL have been conducted in high-resource environments, limiting their generalizability [34]. Furthermore, as Hopkins et al. [28] note, there is considerable heterogeneity in the methodologies used to assess the economic outcomes of IOL, with many studies failing to standardize cost calculations across different healthcare systems and patient populations.

The findings from the reviewed literature provide important considerations for clinical practice and policy-making. Healthcare providers and policymakers should carefully weigh the economic and clinical outcomes of IOL, particularly in resource-constrained environments. Strategies such as membrane sweeping, outpatient balloon catheter inductions, and more conservative expectant management should be considered to reduce unnecessary IOL and its associated costs [28,34]. Policymakers need to create guidelines that prioritize cost-effective methods of IOL, especially in high-risk populations where elective cesareans may be more appropriate to avoid expensive interventions and adverse maternal outcomes [24]. Future policies should also encourage the use of standardized economic models across healthcare settings to better assess the cost-effectiveness of IOL.

Another study by Grobman et al. discusses the economic impact of elective IOL and highlights that it is often associated with increased healthcare costs without significant improvements in health outcomes [24]. The study emphasizes that women undergoing elective IOL are more likely to experience longer hospital stays, increased use of medical interventions such as epidural anesthesia, and higher rates of cesarean delivery, particularly in nulliparous women with an unfavorable cervix. These factors contribute to the overall higher cost of care associated with IOL compared to expectant management. Moreover, the study highlights that, despite the increased resource utilization, there is limited evidence to suggest that elective IOL leads to improved maternal or neonatal outcomes. On the contrary, spontaneous labor is often associated with lower healthcare costs and similar or better health outcomes. The current literature reveals that while IOL may be clinically necessary in certain cases, the widespread use of elective IOL without medical indications contributes to higher healthcare costs without significantly improving health outcomes [32,36,39,41]. To address this issue, alternative strategies such as membrane sweeping or more conservative expectant management should be considered to reduce the need for IOL. 

Two promising techniques, nipple stimulation therapy and membrane sweeping, have shown potential in addressing this need. Nipple stimulation therapy has been proposed as a novel and cost-effective method to induce labor. By encouraging the pulsatile release of endogenous oxytocin, which resembles the natural birth process, nipple stimulation differs from continuous injection of synthetic oxytocin, which may raise obstetric risks and medical expenses. This approach can improve breastfeeding success, which is a critical benefit for the health outcomes of mothers and infants, as well as lessen the need for official labor induction. This approach has the potential to completely transform labor induction procedures, making them more effective and patient-friendly, as evidenced by studies such as the current randomized controlled trial conducted by Tortal et al. [18]. If demonstrated to be successful, nipple stimulation may find widespread use in medical settings, lessening the need for artificial oxytocin and related medical expenses. Membrane sweeping, a different method, considerably lessens the requirement for official induction of labor, particularly in low-resource environments like South Asia. Membrane sweeping encourages spontaneous labor onset and lowers the risk of induction, particularly in multiparous women at 40 weeks or later in pregnancy, per a meta-analysis by Jayasundara et al. [45]. This method has great potential for populations with limited access to cutting-edge healthcare technologies because it is simple to apply in clinical settings. It also adds value to labor management strategies by addressing the growing need for low-cost ways to lower the stillbirth rate among high-risk ethnic groups.

Future research should concentrate on large-scale, multi-ethnic randomized controlled trials (RCTs) to further investigate the effectiveness of these strategies to build on these findings. Special emphasis should be placed on understanding how factors such as parity, period of gestation, and ethnic background influence outcomes. In addition, the integration of such techniques into existing maternity care protocols can be explored to improve maternal and neonatal health outcomes while reducing healthcare costs associated with formal induction methods.

Furthermore, more precise instruments for estimating the risk of cesarean section in women undergoing induction must be developed. Some instruments, like the validated prediction model created by Rossi et al., distinguish between women who are more or less likely to have a cesarean delivery following labor induction based on seven characteristics obtained from the patient's medical record [46]. Healthcare professionals can utilize this free risk calculator, an affordable tool, in conjunction with the Bishop score to counsel women undergoing labor induction and to allocate resources appropriately for those at high risk of cesarean delivery.

## Conclusions

The evidence surrounding IOL highlights both its clinical benefits and economic challenges. While IOL can reduce risks like stillbirth and hypertensive disorders, it is often associated with increased healthcare costs, particularly when it leads to higher cesarean rates, longer hospital stays, and more medical interventions. The cost-effectiveness of IOL varies depending on factors such as gestational age, maternal health, and induction methods, with some techniques like misoprostol being more cost-effective. However, alternatives such as membrane sweeping and expectant management may offer lower-cost solutions in low-risk pregnancies. The literature underscores the importance of tailoring labor management strategies to individual patient needs, balancing clinical benefits with economic sustainability. Future research, especially in resource-constrained environments, is needed to refine guidelines and ensure that IOL is used in a cost-effective and clinically appropriate manner.
